# Usefulness of using pure β-TCP bone substitute (Neobone) in post-orthopedic implant removal procedures

**DOI:** 10.1371/journal.pone.0336404

**Published:** 2025-12-02

**Authors:** Jeong Eun Moon, Young Min Cho, Gwang Chul Lee, Yong Jin Cho

**Affiliations:** 1 Nursing Department, Honam University, Gwangju, Republic of Korea; 2 Department of Medical Science, College of Medicine, Chosun University Graduate School, Gwangju, Republic of Korea; 3 Department of Orthopedic Surgery, Chosun University Hospital, Gwangju, Republic of Korea; Universidade de Trás-os-Montes e Alto Douro: Universidade de Tras-os-Montes e Alto Douro, PORTUGAL

## Abstract

**Purpose:**

The orthopedic implant removal procedure is one of the most commonly performed orthopedic surgeries. This study aimed to compare and report the clinical outcomes between patients who underwent only curettage and those who received pure β-TCP (Neobone^®^) following curettage.

**Methods:**

Between January 1, 2018, and December 31, 2023, patients at our hospital who underwent implant removal and could be followed for over a year were included in the study. The study encompassed 77 cases.

**Results:**

The cohort comprised 40 males (51.9%) and 37 females (48.1%) with an average age of 32.5 ± 21.2 years (range 8–69). There were no significant differences between the groups in terms of sex, age, the interval between the first operation and the orthopedic implant removal procedure, locations of operations, or the orthopedic fixation techniques used initially. However, the group undergoing implant removal with β-TCP (Neobone^®^) bone graft showed statistically significant improvements in performing scar revision procedures simultaneously and in not requiring drain insertion (p = .000).

**Conclusions:**

Compared to the group that only underwent curettage, the group treated with pure β-TCP (Neobone^®^) bone graft required fewer drain placements and more often performed scar revisions simultaneously, facilitating faster healing. While there was no difference in the period for ROM exercises or weight bearing, no refractures occurred in the β-TCP bone substitute group. Hence, the application of pure β-TCP (Neobone^®^) is clinically advantageous.

## Introduction

### Background

Orthopedic implant removal is one of the most frequently performed orthopedic surgeries [1–3]. Once sufficient fusion or bone union has been achieved at a fracture or osteotomy site, including those caused by trauma, the decision to remove the orthopedic implant rests with the patient. The procedure for removing orthopedic implants is comparable in scale to the initial surgery, requires appropriate anesthesia, and involves significant cost and time for both surgery and recovery. Patients often request removal of the implant due to discomfort with retaining a foreign object in their body and desire to improve the appearance of scars from the initial surgery [4,5]. Orthopedic surgeons may advise removing the implant to address concerns related to infection risks, stress shielding fractures, and challenges in managing potential peri-implant fractures that might develop later [2,6–8]. The standard procedure involves removing the orthopedic implant, meticulously curetting the holes where screws or nails were inserted, and mechanically stopping the bleeding. Various techniques have been developed to minimize complications related to orthopedic implant removal, with particular focus on reducing the risk of refracture, a concern for both patients and surgeons [9,10]. The authors have reported favorable clinical outcomes using pure β-TCP (Neobone^®^) as a bone substitute following curettage of benign bone tumors in a previous study [11]. To mitigate complications associated with orthopedic implant removal and concurrently perform scar revision, pure β-TCP (Neobone^®^) was used to fill the resultant holes as an enhanced technique. By integrating these procedures, it was anticipated that the implantation sites for screws or nails would be preserved from becoming occupied by fibrous tissue. Additionally, using pure β-TCP (Neobone^®^) promotes osteoconduction, thereby mitigating the risk of refracture. Furthermore, pure β-TCP (Neobone^®^) functions as a bone plug, arresting bone bleeding and obviating the necessity for inserting a drain, which not only avoids further skin trauma but also meets the patient’s expectations for scar revision and cosmetic improvement.

The purpose of this study was to compare and report the clinical outcomes between a group that underwent only curettage in the orthopedic implant removal procedure and a group that received pure β-TCP (Neobone^®^) insertion after curettage.

### Objectives

The authors aimed to compare the clinical outcomes of the group that underwent a pure β-TCP graft and the group that only underwent curettage during the orthopedic implant removal procedure, and to evaluate the clinical usefulness of the pure β-TCP (Neobone^®^) graft.

## Methods

### Ethics statement

This retrospective study was approved by the Institutional Review Board of Chosun University Hospital (No. CHOSUN-2025-04-009), which waived the requirement for written informed consent due to the retrospective nature of the study.Data were accessed for research purposes between April 23 and May 15, 2025, following approval by the Institutional Review Board (IRB). All methods and procedures were conducted in compliance with pertinent guidelines and regulations.

### Study design, setting and participants

**Fig 1:**
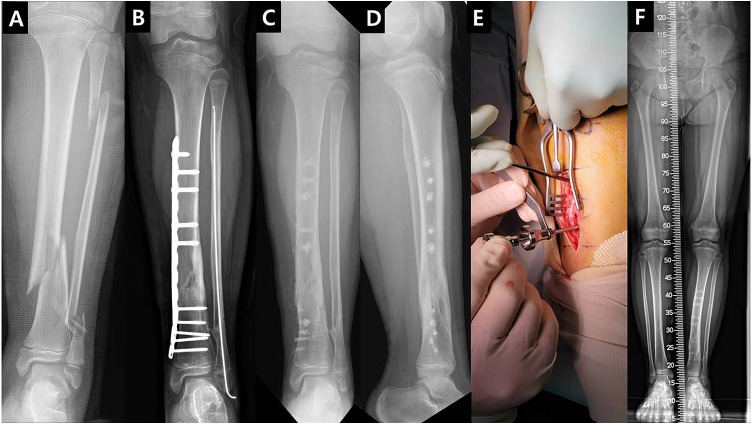
Pediatric tibia implant removal case. A: An 8-year-old child presented with a left tibio-fibular shaft Gust-Anderson Open type I fracture caused by Pedestrian TA. B: For the tibia, plate fixation using the Bridging Technique was performed, and retrograde IM nailing was used for the fibula. C: After determining fracture healing, implant removal was performed, and each screw hole was filled with pure β-TCP (Neobone®) bone substitute graft. D: Lateral x-ray at the same time point showing that the screw holes were effectively filled with pure β-TCP. E: This demonstrates the removal of the screw hole filled with pure β-TCP (Neobone®) bone substitute using a specialized tool. F: Three months post-surgery, the patient walked normally with full weight bearing, and the grafted pure β-TCP (Neobone®) bone substitute demonstrated significant absorption.

From January 1, 2018, to December 31, 2023, patients who underwent implant removal at our hospital were included if they could be followed for more than 1 year. The exclusion criteria were: (1) patients who had K-wires or titanium elastic nails removed, (2) cases where it was not possible to obtain mandatory data that clearly described the factors to be observed, (3) post- operative radiographs were not taken according to protocol. All orthopedic implant removal surgeries were performed by two surgeons (KC Lee, YJ Cho), regardless of who performed the initial surgery. During the study period, a total of 94 orthopedic implant removal operations were conducted at our institution. After excluding 17 cases based on exclusion criteria, 77 cases were included in the study (Fig 1).

**Fig 2:**
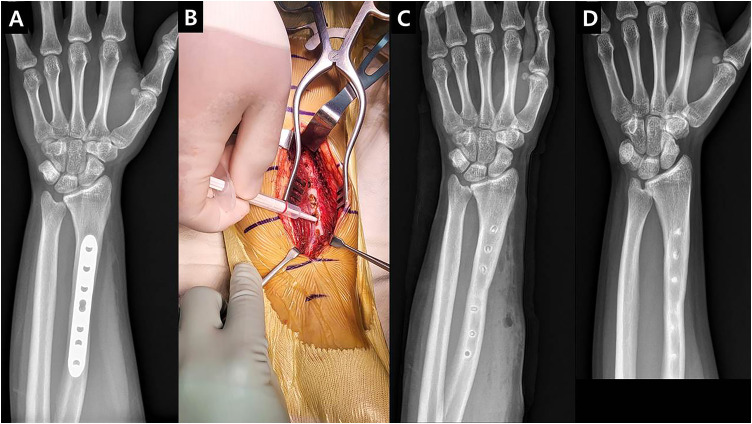
Adult radius implant removal case. A: 21-year-old patient underwent Compressive Plating fixation using LC-DCP after a distal radius corrective osteotomy for 1 year. The osteotomy site was judged completely healed. B: Implant removal and scar revision were performed simultaneously; curettage was performed around the screw hole, followed by insertion of a syringe-type pure β-TCP (Neobone^®^) bone substitute graft. C: Immediately following implant removal surgery, an X-ray reveals the grafted pure β-TCP (Neobone^®^) bone substitute positioned within the hole. D: 1 year after the final surgery, the X-ray shows that the grafted pure β-TCP (Neobone^®^) bone substitute has contributed to host bone formation and achieved cortical healing.

The following factors were investigated: sex, age, interval between the 1st operation and the orthopedic implant removal procedure (months), locations of operations, orthopedic fixation techniques used in the 1st operation (compression plating, bridging plating, intramedullary nailing, screws fixation), the concurrent performance of a scar revision procedure during the 2nd orthopedic implant removal procedure, incomplete removal of the orthopedic implant leaving it inside the patient’s body, whether a drain was implemented, presence of hematoma at the surgical site post-operation, the timing (weeks) of range of motion exercises after the orthopedic implant removal procedure, the timing (weeks) for weight bearing after removal of an orthopedic implant located in the lower extremity, the timing (months) for bone union after the removal of the orthopedic implant, and complications associated with the second orthopedic implant removal procedure (re-fracture, surgical site infection, nerve palsy or injury, heterotopic ossification, etc.) (Fig 2).

### Statistical analysis

Data were input into the computer and analyzed using IBM SPSS Statistics ver. 21.0 (IBM Co., Armonk, NY, USA). Qualitative data were expressed in numbers and percentages. The Kolmogorov-Smirnov test was used to assess the normality of distributions. Quantitative data were described using the mean, standard deviation, and median. A χ^2^ analysis was performed to compare the sex, location, 2st OP technique, complete removal, drain, scar revision, hematoma, ROM exercise, union rates of the different technique. The age, interval, weight bearing duration between different groups of patients was compared using student’s T-test. The level of significance was set at 5%. Summary statistics from the analysis of variance calculations provided 95% prediction limits for measurement errors. A p-value of less than 0.05 was considered statistically significant.

## Results

The study group comprised 40 males (51.9%) and 37 females (48.1%) with a mean age of 32.5 ± 21.2 years (range 8–69). The group that underwent the orthopedic implant removal procedure simultaneously with the insertion of pure β-TCP (Neobone^®^) bone substitute graft included 18 males and 16 females. The mean age in this subgroup was 33.38 ± 21.48 years (range 8–65), and the mean interval between the 1st operation and the orthopedic implant removal procedure was 18.91 ± 7.84 months (range 11–38). In this group, the most common anatomical locations for surgery were the femur/hip/knee with 12 cases (15.6%), followed by the tibia/fibula/ankle with 9 cases (11.7%), the forearm/elbow/wrist with 8 cases (10.4%), and the humerus/shoulder/clavicle with 5 cases (6.5%). Compression plating was the most frequently used orthopedic fixation technique in the 1st operation, occurring in 19 cases (24.7%).

The group that underwent the orthopedic implant removal procedure with only curettage included 22 males and 21 females. The mean age was 31.81 ± 21.31 years (range 8–69), and the mean interval between the 1st operation and the orthopedic implant removal procedure was 20.26 ± 8.89 months (range 11–36). In the same group, the most common anatomical location for surgery was the femur/hip/knee with 17 cases (22.1%), followed by forearm/elbow/wrist with 14 cases (18.2%), tibia/fibula/ankle with 8 cases (10.4%), and humerus/shoulder/clavicle with 4 cases (5.2%). In the same group, compression plating was the most common orthopedic fixation technique used in the first operation, accounting for 23 cases (29.9%). There was no statistical significance in the demographic data between the two groups (Table 1).

**Table 1:** Patient demographic data.

	Pure β-TCP group	Curettage group	*p* value
Sex			0.877
Male	18 (23.4%)	22 (28.6%)	
Female	16 (20.8%)	21 (27.3%)	
Age (years)	33.38 ± 21.48	31.81 ± 21.31	0.750
Interval (months)	18.91 ± 7.84	20.26 ± 8.89	0.491
Location			0.651
Humerus/Shoulder/Clavicle	5 (6.5%)	4 (5.2%)	
Forearm/Elbow/Wrist	8 (10.4%)	14 (18.2%)	
Femur/Hip/Knee	12 (15.6%)	17 (22.1%)	
Tibia/Fibula/Ankle	9 (11.7%)	8 (10.4%)	
1st OP Technique			0.784
Compression Plating	19 (24.7%)	23 (29.9%)	
IM Nailing	6 (7.8%)	7 (9.1%)	
Bridge Plating	7 (9.1%)	12 (15.6%)	
Screw Fixation	2 (2.6%)	1 (1.3%)	

All values are mean ± standard deviation or n (%).

OP, operation; IM, intramedullary.

Regarding incomplete removal of the orthopedic implant, leaving some material inside the patient’s body, no statistically significant difference was observed between the two groups (p = .095). The group that underwent orthopedic implant removal procedure and concurrent pure β-TCP (Neobone^®^) bone substitute graft showed statistically significant outcomes in performing scar revisions simultaneously (p = .000) and avoiding the use of a drain (p = .000).

Three cases of hematoma presence at the surgical site post-surgery were found in the curettage group, but none were found in the pure β-TCP group. However, no statistical significance was found for this (p = .116). No statistical significance was observed between the two groups regarding the timing (weeks) of initiating range of motion exercises after orthopedic implant removal (p = .267), and the timing (weeks) for weight bearing authorization post-removal of the orthopedic implant in the lower extremity (p = .461). In examining the timing (months) of bone union following orthopedic implant removal, in the group that underwent simultaneous orthopedic implant removal and pure β-TCP (Neobone^®^) bone substitute grafting, the occurrences were 23 cases (30.7%) at 3 months, 9 cases (12.0%) at 6 months, and 2 cases (2.7%) at 1 month. In comparison, the group undergoing the procedure with only curettage reported 27 cases (36.0%) at 6 months, followed by 11 cases (14.7%) at 12 months and 3 cases (4.0%) at 3 months (Table 2).

**Table 2:** Subgroup analysis of the 77 patients.

	Pure β-TCP group	Curettage group	*p* value
Complete Removal			0.095
Complete	33 (42.9%)	37 (48.1%)	
Incomplete	1 (1.3%)	6 (7.8%)	
Drain			0.000
Inserted Drain	2 (2.6%)	19 (24.7%)	
No Drain Insertion	32 (41.6%)	24 (31.2%)	
Scar Revision			0.000
Simultaneously	29 (37.7%)	17 (22.1%)	
No Concurrent Procedures	5 (6.5%)	26 (33.8%)	
Hematoma			0.116
Occurred	0 (0%)	3 (3.95)	
Did not develop	34 (44.2%)	40 (51.9%)	
ROM exercises			0.267
Immediate Post-operative	28 (36.4%)	35 (45.4%)	
After 2 weeks	6 (7.8%)	8 (10.4%)	
Weight bearing duration (weeks)	2.19 ± 0.602	2.08 ± 0.400	0.461
Union			0.000
1 month	2 (2.7%)	0 (0%)	
3 months	23 (30.7%)	3 (4.0%)	
6 months	9 (12.0%)	27 (36.0%)	
12 months	0 (0%)	11 (14.7%)	

All values are mean ± standard deviation or n (%).

ROM, range of motion.

Complications associated with the 2nd orthopedic implant removal procedure were noted. Two cases of re-fracture occurred in the group that underwent the procedure with only curettage. The first included an 8-year-old female, who, 12 months after a right femur varization osteotomy and fixation with a dynamic hip screw system, had the implant removed following unionization of the osteotomy site. Immediate post-procedure, both hips underwent ROM exercises. Weight bearing was allowed two weeks post-surgery, and subsequently, a re-fracture at the previous surgical site ensued following a slip. The second case involved a 16-year-old female who underwent compressive plating for a left clavicle mid-shaft fracture. Post-unionization judgement 12 months later, the implant was removed, and re-fracture followed two weeks after the surgery during ROM exercises without any specific trauma. Additionally, a 24-year-old male underwent implant removal 15 months post-compression plating for a humerus shaft fracture. Musculocutaneous nerve palsy in the ipsilateral upper extremity was noted immediately post-procedure, which resolved within 2 weeks. This case occurred in the group that underwent the procedure with simultaneous application of pure β-TCP (Neobone^®^) bone substitute graft. No other instances of surgical site infection or heterotopic ossification were observed.

## Discussion

Orthopedic implants, used for various fixation purposes including repairing fractures caused by trauma, are surgically removed when they are no longer needed [2]. These implants can remain in the body for extended periods, exceeding 50 years, without adverse effects attributable to their design or manufacture [12,13]. Nevertheless, their removal may be necessitated by significant pain, swelling, infection, tendon irritation, tendon rupture, functional impairment, hardware failure, or upon the patient’s request [14]. Haseeb et al. studied 83 patients who underwent hardware removal due to pain, infection, implant failure, or at the patient’s insistence [5]. Of these, 44% experienced complete relief of symptoms while 56% reported partial relief. Brown et al. examined 126 patients after surgical fixation of an ankle fracture; 31% required hardware removal due to pain, and half of these patients showed symptom improvement [1]. Collectively, current studies suggest that about half of the patients undergoing hardware removal for pain or infection experience significant symptom relief [15]. Research comparing rates of hardware removal and associated complications remains limited and varied, thereby complicating interstudy comparisons. Reported complications include infection, impaired wound-healing, nerve damage, ongoing pain, and refracture, with rates varying from 0 ~ 20% [14]. Some research has specifically addressed outcomes after hardware removal for a particular anatomical area; nevertheless, these studies also report a broad spectrum of rates for both general and specific complications, including refracture [9,16,17].

In 2010, a total of 180,000 hardware removal surgeries were performed in Germany, making it the fourth most common surgical procedure in orthopedic surgery, following surgical fracture fixation, arthroscopies, and intervertebral disc interventions [18]. There is an ongoing debate regarding the justification for elective surgical implant removal. The indication for hardware removal is clear in patients with surgical site infections, metal allergies, soft tissue compromise, or failure of osteosynthesis [15]. However, the benefits of relative indications, such as the intended improvement of function, alleviation of foreign body sensation or pain, spatial limitations for future surgical procedures, and the patient’s desire for hardware removal, have not yet been sufficiently proven. In a 2015 study by Reith, which surveyed 332 patients who underwent orthopedic hardware removals at the ankle and wrist joints, 96% of all respondents and 66% of those who experienced subsequent complications indicated they would choose surgical implant removal again [19]. In the same paper, the authors reported that when the indication for hardware removal was pain or limited function, patients experienced a subjective improvement of 95% and 72%, respectively. Several biological effects are associated with metal implants. In particular, metal corrosion poses a significant issue, leading not only to the accumulation of metallic elements in the tissues but also to a deterioration of implant stability [20]. In the pediatric population, this is typically offered routinely as children grow, since the implant could hinder bone remodeling. Additionally, the risk of infection increases at the metal-tissue junction, and allergic reactions may occur due to metal hypersensitivity [21].

Bone grafting is required to manage defects resulting from orthopedic bone tissue removal procedures. Ideally, the bone substitute material should be biodegradable and meet certain requirements, including biocompatibility, adequate initial strength and stiffness, retention of mechanical properties for a sufficient duration to ensure biofunctionality, and nontoxicity of the degradation byproducts [22,23]. β-TCP is one of the most commonly used calcium phosphate compounds for synthetic bone substitute grafts. It is considered safe, nontoxic, free from disease transmission, and unlikely to elicit an immunogenic response [24,25]. Most importantly, it can be used without the risk of morbidity at the donor site. Its synthetic origin ensures complete immune tolerance and eliminates the possibility of viral transmission. β-TCP possesses intrinsic osteoconductive properties, and its handling and storage are relatively easy and safe. Radiographically, the grafted β-TCP demonstrated good incorporation into the host bone and resorption within 12 months [18,26]. This resorption rate is relatively higher than that of other materials.

**Fig 3:**
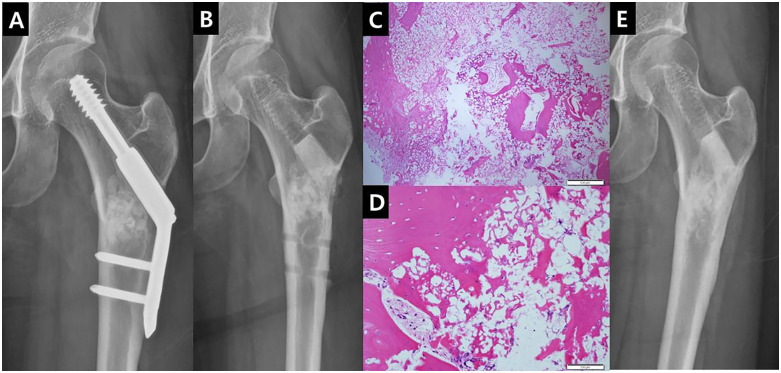
Benign bone tumor fixation case with pathologic reviews. A: This one-year postoperative photograph of a patient, who underwent curettage, pure β-TCP (Neobone^®^) grafting, and 2-hole compressive hip screw fixation for left femur subtrochanteric nonossifying fibroma, demonstrates the healing of the benign bone tumor. B: Implant removal was performed simultaneously with scar revision, and the area around the Lag screw hole was curettaged and filled with a 13 mm cylindrical pure β-TCP (Neobone^®^) bone substitute graft. A biopsy was taken from the previously curettaged surgical site. C: This low-magnification photograph shows dense fibrous collagen tissue on the outer left and the structure of pure β-TCP (Neobone^®^) just inside. Progressing right, the transition from woven bone in the middle to mature bone on the lower right is visible. D: In this high-magnification photograph, the sequence from the pure β-TCP (Neobone^®^) to the bone -forming part from the lower right to the upper left to the mature bone is depicted. The lamellar structure and the small cells, likely osteocytes, are clearly visible. E: An X-ray taken 1 year after the final surgery shows that the grafted pure β-TCP (Neobone^®^) bone substitute has contributed to host bone formation and cortical healing.

The authors have experience in performing curettage, orthopedic implant fixation, and pure β-TCP (Neobone^®^) bone substitute graft for benign bone tumors, subsequently removing orthopedic implants, and analyzing biopsy specimens (Fig 3). Pathological examination revealed that the pure β-TCP (Neobone^®^) bone substitute graft served as a scaffold, promoting the formation of mature bone, osteoid, and osteocytes. The authors concluded that pure β-TCP (Neobone^®^) is effective as a bone plug in the operating room, preventing bone bleeding and hematoma formation without the need for a drainage tube. They anticipated that its use could enhance scar revision outcomes while also preventing refracture.

### Limitations

This study was conducted on a relatively small number of cases and has the limitation of being a retrospective analysis.

## Conclusions

In this study, the authors compared the clinical outcomes between a group that underwent orthopedic implant removal and subsequent filling of the void with pure β-TCP (Neobone^®^) bone substitute, and a group that did not. The group treated with pure β-TCP (Neobone^®^) bone substitute had reduced drain placement, simultaneous scar revision procedures, and faster bone union compared to the group that only underwent curettage. There was no difference in the ROM exercise period or weight bearing period between the two groups, but refracture did not occur in the pure β-TCP (Neobone^®^) bone substitute group, indicating its clinical utility.
